# Supplementary material for: Cost Evaluation During Decision-Making in Patients at Early Stages of Psychosis
Source: Comput Psychiatr. 2019 Feb;3:18–39. doi: 10.1162/cpsy_a_00020 (PMC6436576; doi:10.1162/cpsy_a_00020)
Supplement: Supplementary file 1 [file cpsy-03-18-s001.pdf]

Ermakova, A. O., Gileadi, N., Knolle, F., Justicia, A., Anderson, R., Fletcher, P. C., Moutoussis, M., & Murray, G. K. (2019). Appendix: Expectation-Maximization. Supplement to "Cost evaluation during decision-making in patients at early stages of psychosis." *Computational Psychiatry*, *3,* 18–39. https://doi.org/10.1162/cpsy_a_00020

Appendix: Expectation-Maximization

Expectation-Maximization adjusts the participant-level parameter estimates and the group-level parameter estimates to make them maximally consistent with the data (E) and with each other (M). The group level distributions are gamma-shaped. For the purposes of algebraic manipulation, it is convenient to parameterise these in terms of a so-called shape parameter κ and a scale parameter θ, but can also they can be equivalently described by their mean $m=\kappa\theta$ and variance $\sigma^2 = \kappa\theta^2$. We report values for mean and variance as we believe they are more intuitively understandable by clinical researchers but use the shape-scale notation in this appendix for algebraic clarity. The prior probability for each participant's parameters under the group parameters in our generative statistical model G (not to be confused with *G* for 'gold') is:

$$p(CS, T; G) = f(CS;\ \kappa_{CS}, \theta_{CS}) f(T;\ \kappa_T, \theta_T) \qquad \text{Equation A1}$$

At the participant level, we denote the (empirical) posterior beliefs about the parameters of each participant j , furnishing data d_j as Q[T, CS; d_j]. Again Q here not to be confused with action values. We write $v=\{T,CS\}$ and the probability that the participant made all the decisions they did under equation 5 as $P[d_j \mid v; G]$. Now, we seek to maximise the aforementioned consistency by minimizing the free energy of the entire model, given the data:

$$\begin{aligned} F(Q,G) &= \langle \textstyle\sum_v Q[v; d_j](\ln(p[v, d_j; G]/Q[v; d_j]))\rangle_j \\ &= \langle \textstyle\sum_v Q[v; d_j](\ln P[d_j|v; G] + \ln(p[v; G]) - \ln(Q[v; d_j]))\rangle_j \end{aligned} \qquad \text{Equation A2}$$

Substituting Eq. 1a gives

$$\begin{aligned} F(Q,G) = \langle \sum_{CS,\tau} Q[CS, \tau; d_j](\ln P[d_j|CS, \tau] + \\ \ln(f[CS; \kappa_{CS}, \theta_{CS}]) + \ln(f[\tau; \kappa_\tau, \theta_\tau]) \\ - \ln(Q[CS, \tau; d_j]))\rangle_j \end{aligned} \qquad \text{Equation A3}$$

In the maximisation phase, we keep the form of all Q[T, CS; d_j] fixed and differentiate Eq. A3 w.r.t. G, that is, $\kappa_{CS}, \theta_{CS}, \kappa_T, \theta_T$. We can derive:

$$\frac{\partial F(Q, G(\kappa_\tau, \theta_\tau))}{\partial \kappa_\tau} = \langle \sum_{CS,\tau} Q[CS, \tau; d_j](\ln(\tau) - ln(\theta_\tau) - \Psi_0(\kappa_\tau))\rangle_j \quad \text{Equation A4}$$

Where Ψ_0 is the digamma function. In an analogous fashion,

$$\frac{\partial F(Q, G(\kappa_\tau, \theta_\tau))}{\partial \theta_\tau} = \langle \sum_{CS,\tau} Q[CS, \tau; d_j](\tau/{\theta_\tau}^2 - \kappa_\tau/\theta_\tau)\rangle_j \quad \text{Equation A5}$$

Setting Eq. A5 equal to zero and using integrals instead of sums, as the parameters are continuous, gives for the group mean T_m :

$$\kappa_\tau \theta_\tau = \tau_\mu = \langle \iint_{CS,\tau} Q[CS, \tau; d_j]\ \tau\ dCS\ d\tau\rangle_j := I_1 \quad \text{Equation A6}$$

We evaluated this expectation numerically and used it to express $\kappa_T = I_1 / \theta_T$. We then set Eq. A4 equal to zero and obtained:

$$I_2 := \langle \sum_{CS,\tau} Q[CS, \tau; d_j] \ln(\tau)\rangle_j = (\ln(\theta_\tau + \Psi_0(\kappa_\tau))\langle \sum_{CS,\tau} Q[CS, \tau; d_j]\rangle_j \quad \text{Equation A7}$$

$$\Rightarrow I_2 = \ln(\frac{I_1}{\kappa_\tau}) + \Psi_0(\kappa_\tau)$$

$$\Rightarrow \ln(I_1) - I_2 = ln(\kappa_\tau) - \Psi_0(\kappa_\tau)$$

Again, I_2 is integrated numerically and Eq. A7 solved for κ_T by refining its standard approximate algebraic solution numerically.

The Expectation step can proceed much more straightforwardly, in terms of algebra, as Bayes theorem gives:

$$Q[CS, \tau; d_j] = P[CS, \tau | d_j; G] = \frac{P[d_j|CS, \tau] f(CS; \kappa_{CS}, \theta_{CS}) f(\tau; \kappa_\tau, \theta_\tau)}{\iint_{CS,\tau} P[d_j|CS, \tau] f(CS; \kappa_{CS}, \theta_{CS}) f(\tau; \kappa_\tau, \theta_\tau)\ dCS\ d\tau}$$

Equation A8

In this low-dimensional parameter space the denominator was numerically tractable using Monte-Carlo integration, but for high dimensional models algebraic approximations would be needed.

Once EM has converged, equation A8 gives the probability distributions for individual participants, which can themselves be expressed in terms of shape and scale, or mean and variance.
